# Mining Electronic Health Records to Promote the Reach of Digital Interventions for Cancer Prevention Through Proactive Electronic Outreach: Protocol for the Mixed Methods OptiMine Study

**DOI:** 10.2196/23669

**Published:** 2020-12-31

**Authors:** Michael S Amato, Sherine El-Toukhy, Lorien C Abroms, Henry Goodfellow, Alex T Ramsey, Tracey Brown, Helena Jopling, Zarnie Khadjesari

**Affiliations:** 1 Truth Initiative Washington DC, DC United States; 2 College of Medicine and Science Mayo Clinic Rochester, MN United States; 3 Division of Intramural Research The National Institute on Minority Health and Health Disparities The National Institutes of Health Bethesda, MD United States; 4 Department of Prevention and Community Health Milken Institute School of Public Health The George Washington University Washington DC, DC United States; 5 Department of Primary Care and Population Health University College London London United Kingdom; 6 Department of Psychiatry Washington University School of Medicine St Louis, MO United States; 7 Behavioural and Implementation Science research group School of Health Sciences University of East Anglia Norwich United Kingdom; 8 West Suffolk NHS Foundation Trust Bury St Edmunds United Kingdom

**Keywords:** EHR, electronic health record, smoking cessation, alcohol reduction, proactive outreach, proactive messages, electronic messages

## Abstract

**Background:**

Digital behavior change interventions have demonstrated effectiveness for smoking cessation and reducing alcohol intake, which ultimately reduce cancer risk. Leveraging electronic health records (EHR) to identify at-risk patients and increasing the reach of digital interventions through proactive electronic outreach provide a novel approach that may increase the number of individuals who engage with evidence-based treatment.

**Objective:**

This study aims to increase the reach of digital behavior change interventions by implementing a proactive electronic message system for smoking cessation and alcohol reduction among a large, at-risk population identified through an acute hospital EHR.

**Methods:**

This protocol describes a 3-phase, mixed-methods implementation study to assess the acceptability, feasibility, and reach of a proactive electronic message system to digital interventions using a hospital’s EHR system to identify eligible patients. In Phase 1, we will conduct focus group discussions with patients and hospital staff to assess the overall acceptability of the electronic message system. In Phase 2, we will conduct a descriptive analysis of the patient population in the hospital EHR regarding target risk behaviors and other person-level characteristics to determine the project’s feasibility and potential reach. In Phase 3, we will send proactive messages to patients identified as smokers or risky drinkers. Messages will encourage and provide access to behavior change mobile apps via an embedded link; the primary outcome will be the proportion of participants who click on the link to access information about the apps.

**Results:**

At the time of initial protocol submission, data collection was complete, but analysis had not begun. This study was funded by Cancer Research UK from April 2019 to March 2020. Health Research Authority approval was granted in June 2019.

**Conclusions:**

Increasing the reach of digital behavior change interventions can improve population health by reducing the burden of preventable death and disease.

**International Registered Report Identifier (IRRID):**

DERR1-10.2196/23669

## Introduction

Cigarette smoking and risky alcohol consumption, both modifiable behaviors, are among the leading causes of cancer and other diseases [1,2]. Increasing the reach of evidence-based interventions that effectively help people quit smoking and drink less can improve population health by reducing the burden of preventable death and disease.

In 2018, 14.7% of adults 18 years and older in the United Kingdom smoked cigarettes (16.5% among men vs 13.0% among women), which is equivalent to roughly 7.2 million people [3]. Although cigarette smoking has steadily declined since 2011, disparities exist in smoking rates by age, socioeconomic status, employment status, and ethnicity, in addition to location-based disparities [3]. In England, smoking led to 77,800 deaths and 489,300 hospital admissions in 2018 [3]. By 2035, reducing tobacco use to under 5% across all socioeconomic groups in the United Kingdom would prevent 35,901 new cases of tobacco-related cancers, 28,997 cases of chronic obstructive pulmonary disease, 24,854 cases of stroke, and 7594 cases of coronary heart disease [4].

Alcohol consumption above 14 drinks/week for men and women, referred to herein as risky drinking, is known to increase risk of alcohol-related harm [5]. In the United Kingdom, 28% of men and 14% of women, accounting for 21% of adults 16 years and older, drank alcohol at risky levels in 2019 [6,7]. In 2018, there were 7551 alcohol-related deaths in the United Kingdom, which is equivalent to 11.9 deaths/100,000 population [8]. Similar to cigarette smoking, disparities exist in alcohol-related deaths by gender, age, location, socioeconomic status, employment status, race, and ethnicity [8]. Further, the comorbidity of tobacco and alcohol use is well documented and results in higher dependency and exacerbated adverse health consequences compared to those experienced by sole users of either tobacco or alcohol [9].

Scalable public health interventions are necessary to reduce the impact of tobacco use and risky drinking. Digital health interventions have demonstrated effectiveness for promoting smoking cessation and reducing alcohol use [10-15]. The availability of digital health interventions has rapidly increased over the past decade fueled by scientific interest, commercial investment, and public demand for these interventions [16-18].They can overcome psychological and logistical barriers (eg, stigma, cost, time) that impede the reach of traditional behavioral interventions (eg, in-person or group counselling).Their demonstrated effectiveness, combined with their scalability to reach large populations at a relatively low incremental cost per user, has the potential to achieve high population health impact [10,19,20].

One approach for distributing digital interventions to reach members of the population is to leverage electronic health records (EHRs). Originally intended to replace paper documents on patients’ medical histories, EHRs are now widespread in health care systems, driven by evidence of their positive impact on health care quality and efficiency [21,22]. In the United Kingdom, adoption of EHRs in general practices is nearing saturation, whereas adoption in secondary care is not as widespread. [23]. Studies have documented the use of EHR-based electronic communication with patients to improve service outcomes such as attendance rates, vaccination rates, and cancer screening [24-27]. Similarly, studies have documented benefits of EHR-based reminders, decision support systems, performance feedback, and easy referral to services in supporting physicians’ duties in screening and treating patients [28-40].

Previous studies have used EHRs to identify subgroups of patients and then proactively connect them with counselling, removing a barrier to reach by eliminating the need for clinician referral [38,39,41,42]. For example, Haas and colleagues [41] identified smokers of low socioeconomic status through a hospital EHR and contacted them using interactive voice response to deliver treatment. They found that patients who participated in proactively offered telephone counselling were more likely to quit smoking than those who did not [41]. Similarly, Fu et al [42] found that offering tobacco cessation treatment to smoking veterans identified through an EHR increased quit rates compared to participants who received only usual care. Taken together, these results suggest that proactive outreach of behavioral interventions to patients who have been identified through EHRs is an acceptable and effective strategy for increasing the reach of health services. Less evidence exists regarding the acceptability or effectiveness of proactively offering patients fully digital interventions. In one study, Abroms et al [43] found that, among smokers, a digital treatment offering was acceptable and had higher reach than one that involved phone counselling. If acceptable, such an approach would leverage the scalability of digital interventions to deliver treatment at lower costs than approaches that rely exclusively or in part on traditional in-person or telephone counselling.

This 3-phase, mixed methods implementation study aims to implement and assess the acceptability, feasibility, and reach of an EHR-based system for identifying at-risk adults and promoting digital interventions. The design is guided by the taxonomy of implementation outcomes described by Proctor et al [44]. Specifically, adults who smoke cigarettes or drink alcohol at risky levels will be identified from a hospital’s EHR and sent proactive electronic messages that promote mobile apps endorsed by Public Health England for smoking cessation and alcohol drinking reduction: “SmokeFree” [45] and “Drink Free Days” [46]. In Phase 1, formative research on the acceptability of the electronic message system will be conducted through focus groups. In Phase 2, feasibility of the approach will be assessed through descriptive analysis of patient records in the EHR. Finally, in Phase 3, patients identified through the EHR will receive a message via email, text message (SMS), or patient portal that contains a link to access the app appropriate for their risk profile. The primary outcome of Phase 3 is the proportion of patients who follow an embedded link in the message to access the app. Our hypothesis is that proactive electronic messages will engage a clinically meaningful proportion of at-risk patients in digital interventions for health behavior change, defined here as ≥5%.

## Methods

### Setting

The project will take place at West Suffolk NHS Foundation Trust (WSFT), a provider of acute and community services to a population of around 300,000 people based in Bury St. Edmunds, Suffolk, United Kingdom. WSFT is one of 17 acute National Health Service (NHS) Trusts that are internationally recognized providers of exceptional and efficient NHS care via world-class digital technology and information, known as a Global Digital Exemplar [47].

The WSFT EHR system—Cerner Millenium, locally named “eCare”—includes records for all outpatients and inpatients registered with the hospital. Smoking and alcohol data are captured in the Activity of Daily Living Nursing assessment and a bespoke Lifestyle Screening form completed by nurses and doctors at the time of admission to hospital [48]. The EHR also contains contact information, demographics, and other health data such as chronic disease status.

### Phase 1: Acceptability

#### Overview

Acceptability has been defined as “the demonstrable willingness within a user group to employ information technology for the tasks it is designed to support” [49]. Focus groups are a qualitative method to collect in-depth information about participants’ opinions, attitudes, and beliefs around a specific topic [50] and are thus suited for acceptability studies. In this phase, we will conduct 6 focus groups onsite at WSFT to measure patient and stakeholder acceptability and preferences of using EHRs to identify at-risk patients and send them electronic messages that promote behavior change mobile apps.

#### Participants

Participants, both patients and hospital staff, must be at least 18 years of age. Patients must self-identify as smokers and/or individuals who regularly consume alcohol. We will recruit participants who drink alcohol at any level (not just risky levels) to avoid alienating patients who do not recognize they are drinking at risky levels or those who do not wish to identify as such. We will attempt to balance participants by gender and age.

#### Data Collection

Topic guides will be based on Perceived Attributes of eHealth Innovations [51] and the diffusion of innovation theory [52], which can explain and predict an intervention’s acceptability and potential for adoption [51,53]. Specifically, we will focus the topic guide on 3 constructs central to the Perceived Attributes of eHealth Innovations and diffusion of innovation theory—relative advantage, complexity, and compatibility. These 3 attributes were selected for their alignment with the larger construct of acceptability (our Phase 1 focal point), and we will focus equally on these 3 attributes in terms of question intensity and analysis. To the extent that participant discussions are responsive to these attributes, we do not intend to prioritize one attribute over another.

We will conduct 3 focus groups with patients: one with patients who smoke tobacco, one with patients who drink alcohol, and a third with patients who smoke tobacco and drink alcohol. In addition to questions about the acceptability of the electronic message system, we will ask patients about their preferences for message modality (email, SMS, online patient portal, other), opinions about the content of messages (see Multimedia Appendix 1 for sample messages), and any potential unanticipated consequences.

We will also conduct 3 focus groups with hospital staff: one with health care professionals who undertake lifestyle screening, one with EHR administrative staff (eg, information analysts, information technologists, communications staff), and a third with senior managers in the eCare team. In addition to questions about the acceptability of the messages, we will ask hospital staff to identify any technical, legal, privacy, ethical, or medical challenges or concerns they foresee in implementing the proposed system within the hospital’s EHR.

#### Procedures

We will recruit patients via the WSFT website and through a volunteer organization that supports research studies at the hospital. Hospital staff will be recruited via intranet, email newsletter, word of mouth, and a monthly corporate briefing. Patients and staff members who express interest in the focus groups will be emailed an information sheet and a consent form. Participants must sign the consent forms prior to any research activity. Participants in the patient focus groups will complete a brief, anonymous, demographic form similar to the demographic characteristics data recorded in the EHR (see Phase 2). Patients will be offered a £25 (US $34) voucher as a token of appreciation.

Each focus group will include approximately 6-8 participants and last approximately an hour. All focus groups will be audio-recorded. A professional transcription service will transcribe the audio recording verbatim, and all personal identifying information will be removed.

#### Analytic Plan

 Qualitative coding software NVivo will be used to organize the themes in the transcripts [54]. Focus groups will be coded by one researcher, and coding will be cross-checked by a second independent researcher. Researchers will meet regularly to discuss the coding and any discrepancies will be resolved collaboratively. Framework analysis will be used to analyze the data, guided by the Perceived Attributes of eHealth Innovations [51]. We will also use inductive analysis where themes will be identified from the data [55]. Data from the focus groups will inform messaging decisions in Phase 3. For example, results from the focus groups will determine whether messages are sent via SMS, email, or patient health portal and the framing of the message content in order to maximize acceptability and minimize any concerns.

### Phase 2: Feasibility

#### Overview and Data Collection

Feasibility assesses whether the methods and procedures of a proposed study will work before implementing it on a large scale [56]. We will work with hospital information analysts to identify the proportions of patients who smoke, drink alcohol at risky levels, or both in the hospital’s EHR. Obtaining information about the patient population will inform the planned segmentation in Phase 3. Specifically, we will mine the EHR for the following key data fields: risk profile, contact information, demographic characteristics, chronic or past health conditions, and screening recency. The risk profile is categorized as (1) exclusive smokers, (2) exclusive risky alcohol drinkers, and (3) dual smokers and drinkers. Contact information includes an email address, a mobile phone number, or a record of access to the online patient portal. Demographic characteristics include gender, age, ethnicity, and level of economic deprivation (Index of Multiple Deprivation using post code). Chronic or past health conditions include hypertension, high cholesterol, heart disease, chronic kidney disease, stroke, chronic obstructive pulmonary disorder, asthma, diabetes, dementia, cancer, arthritis, schizophrenia, bipolar and/or other psychosis, and depression and/or anxiety. Screening recency is the date on which tobacco and alcohol use status was most recently assessed.

#### Analytic Plan

We will use descriptive statistics to tabulate the frequencies and percentages of patients with valid data in each of the aforementioned key fields. Availability of contact information, demographics, health conditions, and screening recency will be stratified by risk profile. Distributions will be compared with chi-squared tests and logistic regression analyses. No personally identifiable information nor patient-level data will be available to the study team. The study team will provide table shells to the hospital-based information analysts, who will aggregate the data onsite and return deidentified results to the study team, consistent with hospital policies. Findings from Phase 2 will inform the viability of the proposed 3 risk profile groups to be examined in Phase 3. A sample size of 383 will be needed for each risk profile to produce a 95% confidence interval of ±5%, assuming a population proportion of 50% and a population size of 100,000 [57]. While we plan to conduct Phase 3 regardless of the true EHR population size as revealed in Phase 2, this statistic provides a benchmark for interpreting results. Furthermore, although not an outcome for the proposed study, findings from Phase 2 will be shared with hospital staff to identify any potential gaps in completeness of records and to suggest potential changes to hospital protocols to improve their completeness.

### Phase 3: Reach

#### Overview

The reach of a public health intervention can be defined as “The proportion and representativeness of individuals who are willing to participatein a given initiative, intervention, or program.” [58]. In this phase, we will send proactive electronic messages to at-risk patients who have visited the hospital in the past year, encouraging them to access behavior change apps that support smoking cessation and alcohol reduction. The primary outcome will be the click rate on the link embedded in these messages. We will tailor messages to each participant’s risk profile (ie, smoker, risky drinker).

#### Participants

All identified smokers and risky drinkers for whom contact information is available will be eligible to receive messages, except for patients <18 years old; patients on the End of Life Pathway; pregnant women, as their data are held in a separate database and governed by separate hospital policies; and patients who have opted out of receiving messages from the hospital.

#### Procedures

The final content and modality of the messages will be based on the acceptability and feasibility findings from Phase 1, and the final number of participants will be based on the feasibility findings from Phase 2. Eligible patients will be sent messages containing a link to a webpage hosted by the NHS where participants can download a free behavior change app relevant to their risk profile. These apps include “SmokeFree” and *“*Drink Free Days,” both of which are promoted by Public Health England as part of their “OneYou” campaign [45,46]. The apps are available for iOS and Android devices.

A data privacy impact assessment has been performed to assess the risk posed by processing patients’ data in order to send communications promoting behavior change apps [59]. Each participant will receive at least one initial message and another reminder message if they do not click the link in the initial message; the total number of messages will be determined by the results of Phase 1. To capture and track click rates, each message-embedded link will contain a tracking code that is unique to its recipient. Information technologists at the hospital will use the tracking codes to identify which participants clicked through to access the NHS webpage.

Approximately 1 week after the intervention messages have been sent, participants will receive a message inviting them to complete an anonymous follow-up survey. The survey will collect information on their experience of receiving the message, to further inform acceptability. As in Phase 1, survey items in Phase 3 will be based on the Perceived Attributes of eHealth Innovations and will examine 3 acceptability constructs: relative advantage, simplicity, and compatibility [51]. Patients who complete the survey will be entered into a prize draw to win £250 (US $338) high street vouchers.

#### Measures

With the exception of the primary outcome (ie, click rate of the link), all study measures are defined within the WSFT EHR system. For behavioral risk status, smokers are defined by a single item labelled “Does the patient smoke?”, and risky drinkers are defined by the hospital as those with an Alcohol Use Disorders Identification Test–Consumption version (AUDIT-C) score between 5 and 10 [60]. Demographics include gender, age, ethnicity, and Index of Multiple Deprivation. Health conditions include hypertension, high cholesterol, heart disease, chronic kidney disease, stroke, chronic obstructive pulmonary disorder, asthma, diabetes, dementia, cancer, arthritis, schizophrenia, bipolar and/or other psychosis, and depression and/or anxiety. The primary outcome for Phase 3 is the click-through rate of the links embedded in the messages. In other words, the outcome is the proportion of participants who click the message link to access a behavior change app, with the numerator being the number of individuals who clicked the link and the denominator being the number of individuals who received the message.

#### Analytic Plan

We will use logistic regression analysis to model the likelihood of clicking on the message-embedded link within each risk group, with demographics and health condition statuses entered as covariates. The logistic regression approach will support comparison of effect sizes as the relative risk, a metric that is familiar and easily interpretable across the health sciences. Additional analyses will explore the click rate stratified by risk group and sociodemographic characteristics to investigate health disparities, if any.

We determined a priori that 5% would be a clinically meaningful proportion of users clicking on the message-embedded link. This value was selected based on feasibility determined by prior research [39]. Although relatively modest in absolute terms, 5% of the total number of smokers or risky alcohol drinkers within the NHS EHRs would represent many people. We hypothesize that 5% is attainable based on similar studies in which patients identified through an EHR responded to a quitline referral [39,43] or proactive telephone calls offering telephonic coaching [41] and on email marketing campaign industry standards of click-through rates of 2%-5% [61]. This includes those whose mobile phone numbers are invalid (eg, message undeliverable) and therefore unable to receive the messages.

### Ethics Approval

**Figure 1:**
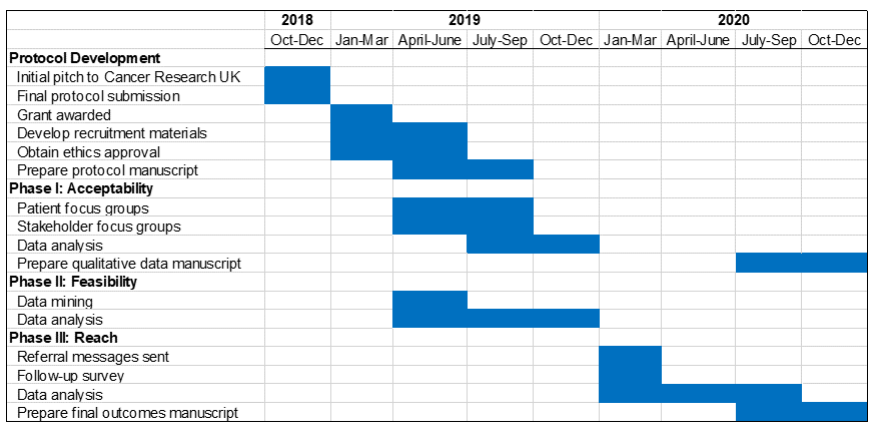
Gantt chart of study timeline.

We will seek ethical approval from the NHS Research Ethics Committee and the Health Research Authority. All communications with patients in Phase 3 will come from the hospital, rather than the study team, and only patients who have consented to receive health communications from the hospital are eligible to be included in the study. We have already received authorization to use EHR data from the WSFT Information Governance Team via a data protection impact assessment form [59] (approval date: September 20, 2019). The study timeline is presented in a Gantt chart (see Figure 1).

## Results

This study was funded by Cancer Research UK from April 2019 to March 2020. NHS Research Ethics Committee and Health Research Authority approvals were granted in June 2019. At the time of the original submission of this protocol manuscript, data collection was complete, but analyses had not begun. Analysis of phase 3 data was extended beyond the end of the study, due to prioritization of COVID-19 activity.

## Discussion

Increasing the reach of digital behavioral health interventions can improve population health by reducing the burden of preventable death and disease. This 3-phase study protocol describes an implementation strategy that aims to increase exposure to digital-based treatment for smoking cessation and alcohol reduction among a large, at-risk population identified through a hospital’s EHR. While future research is needed to assess what proportion of users who click a link subsequently enroll in treatment, this study will provide empirical estimates of an important first step on that pathway. Specifically, we aim to use electronic communications to promote Public Health England’s behavior change mobile apps to secondary care patients who smoke or drink alcohol at risky levels. The implementation strategy [62] is designed to be scalable, such that the study protocol can be readily and economically translated to an automated system for immediate implementation within a hospital EHR system.

This study is one of the first to integrate digital behavior change interventions with an EHR system. This study will contribute to the literature by providing valuable data on the acceptability, feasibility, and reach of such integration. The study will advance the dissemination of digital health interventions, expand the use of EHR beyond the individual patient, and provide a model for implementation and adaptation to other health care systems. Finally, a fully automated screening and proactive outreach system to digital behavioral interventions will preserve human resources for cases that need special attention, such as patients who need personal follow-up calls to enroll in behavioral change interventions or patients who may require in-person behavioral counselling such as smokers from special populations such as HIV patients or those with mental illness.

Future directions include follow-up studies to determine optimal message content, frequency, and other communication meta parameters to yield higher click rates. Similarly, follow-up studies should explore group differences in patient preferences for engaging with electronic communications about behavior change support. Other future directions include deeper integration of interventions with EHRs, for example dynamically tailored interventions based on clinician-initiated changes to a patient’s EHR or reporting information bidirectionally from a digital intervention back to the EHR for clinician review. Finally, follow-up studies on implementation fidelity and adaptability to health care systems nationally and worldwide are needed.

### Limitations

The proposed study takes place at a Global Digital Exemplar Trust, a world leader in digital technology and health records. This infrastructure might not be available at other hospitals. The intervention is based on the use of electronic communication (email, SMS, online patient portal) and directs patients to download mobile apps for behavioral interventions; patients who are not connected to the internet or do not own smartphones are unlikely to benefit from such approaches. Race/ethnicity and socioeconomic status are important determinants of smoking and alcohol drinking behaviors; however, the sample for the current study is limited by the hospital population.

### Conclusion

This implementation study will add valuable insights to sparse literature on using EHRs to expand the reach of digital interventions for cancer prevention. The ultimate goal of the research is to reduce cancer incidence on a population level by addressing cancer risk behaviors such as cigarette smoking and drinking alcohol at risky levels. Results will guide future studies on wider-scale implementation.

## Appendix

Multimedia Appendix 1 Focus group example messages.

## Abbreviations

EHR: electronic health record
NHS: National Health Service
WSFT: West Suffolk NHS Foundation Trust
