# Supplementary material for: Mining Electronic Health Records to Promote the Reach of Digital Interventions for Cancer Prevention Through Proactive Electronic Outreach: Protocol for the Mixed Methods OptiMine Study
Source: JMIR Res Protoc. 2020 Dec 31;9(12):e23669. doi: 10.2196/23669 (PMC7808893; doi:10.2196/23669)

Are you thinking about stopping smoking? Did you know there is a free app on the NHS that could help? Please click below if interested:

<https://www.nhs.uk/oneyou/apps>

Want to stop smoking or reduce drinking? The NHS has free apps that could help you:

<https://www.nhs.uk/oneyou/apps/>

Are you thinking about reducing your drinking? <https://www.nhs.uk/oneyou/apps/>

Hello. This is West Suffolk Hospital. We think this app will help you to stay smokefree:

<https://www.nhs.uk/oneyou/apps/>

Hello. This is West Suffolk Hospital. We advise you to reduce your drinking. Please click: <https://www.nhs.uk/oneyou/apps/>

Hello this is West Suffolk Hospital. Smokers can lose an average of 10 years of life. If you are still smoking click the below to help support you to quit:

<https://www.nhs.uk/oneyou/apps/>

This NHS smokefree app can help you to stop smoking by providing daily support and motivation. If you stay smokefree for the 4-week programme you’re up to 5 times more likely to quit for good:

<https://www.nhs.uk/oneyou/apps/>

This NHS Drink Free Days app is a simple way to track the days you drink alcohol. Feel healthier, lose weight and save money- choose days to take off drinking and get practical daily support to help you stick to it:

<https://www.nhs.uk/oneyou/apps/>

Reducing your drinking is hard but you can do it!

<https://www.nhs.uk/oneyou/apps/>

It’s easier to quit smoking with support. Click this link now!

<https://www.nhs.uk/oneyou/apps/>

Quitting smoking is the most important thing you can do for your health. Thousands quit every day. You can too!

<https://www.nhs.uk/oneyou/apps/>

Hi it’s West Suffolk Hospital with a friendly reminder that it’s easier to quit with this app:

<https://www.nhs.uk/oneyou/apps/>

Or text STOP to cancel.

When the link is clicked…


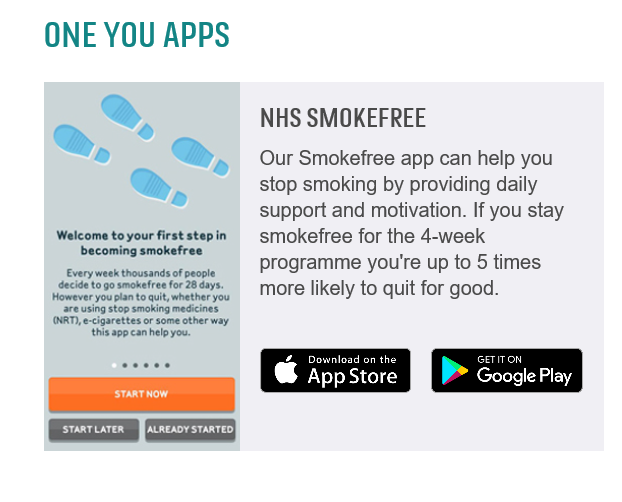


When the link is clicked…


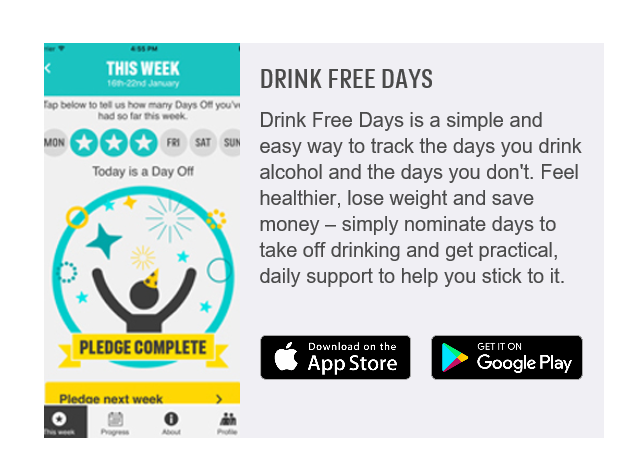

Supplement: Multimedia Appendix 1 [file resprot_v9i12e23669_app1.docx]
